# Comparison of cross sectional optical coherence tomography images of elevated optic nerve heads across acquisition devices and scan protocols

**DOI:** 10.1186/s40662-018-0112-3

**Published:** 2018-07-14

**Authors:** Megh D. Patel, Fareshta Khushzad, Heather E. Moss

**Affiliations:** 1 Department of Ophthalmology, Stanford University, 2370 Watson Court, suite 200, Palo Alto, CA 94303 USA; 2 Department of Neurology and Neurological Sciences, Stanford University, 2370 Watson Court, suite 200, Palo Alto, CA 94303 USA

**Keywords:** Optic nerve head, Optical coherence tomography, Bruch’s membrane, Inner limiting membrane, Comparison

## Abstract

**Background:**

Optic nerve head measurements extracted from optical coherence tomography (OCT) show promise for monitoring clinical conditions with elevated optic nerve heads. The aim of this study is to compare reliability within and between raters and between image acquisition devices of optic nerve measurements derived from OCT scans in eyes with varying degrees of optic nerve elevation.

**Methods:**

Wide angle line scans and narrow angle radial scans through optic nerve heads were obtained using three spectral domain(SD) OCT devices on 5 subjects (6 swollen optic nerves, 4 normal optic nerves). Three raters independently semi-manually segmented the internal limiting membrane(ILM) and Bruch’s membrane(BM) on each scan using customized software. One rater segmented each scan twice. Segmentations were qualitatively and quantitatively compared. Inter-rater, intra-rater and inter-device reliability was assessed for the optic nerve cross sectional area calculated from the ILM and BM segmentations using intraclass correlation coefficients and graphical comparison.

**Results:**

Line scans from all devices were qualitatively similar. Radial scans for which frame rate could not be adjusted were of lower quality. Intra-rater reliability for segmentation and optic nerve cross sectional area was better than inter-rater reliability, which was better than inter-device reliability, though all ICC exceeded 0.95. Reliability was not impacted by the degree of optic nerve elevation.

**Conclusions:**

SD-OCT devices acquired similar quality scans of the optic nerve head, with choice of scan protocol affecting the quality. For image derived markers, variability between devices was greater than that attributable to inter and intra-rater differences.

## Background

Optical coherence tomography (OCT) and the micron level cross-sectional imaging of the retina that it provides is becoming ubiquitous in clinical ophthalmology. One clinical application in the field of neuro-ophthalmology is visualization of the swollen optic nerve in cross section, which allows quantitative measurement of the nerve head contours for purposes of diagnosing and monitoring anterior optic nerve abnormalities including papilledema due to elevated intracranial pressure (ICP), anterior ischemic optic neuropathies, optic nerve head drusen and anterior optic neuritis. Optic nerve head volume or cross sectional area which is increased by drusen deposits, or axoplasmic stasis caused by ischemia, inflammation or retrobulbar optic nerve compression may be relevant clinical metrics [1]. For example, in cases of papilledema, optic nerve head volume correlates with the qualitative Frisen severity scale [2] and resolves in association with treatment of elevated ICP [3]. The contour of the ocular globe around the optic nerve, which assumes a flatter contour in states of elevated ICP, may also be a useful clinical metric for monitoring ICP [4, 5] and for differentiating causes of optic nerve head swelling [6]. Both of these parameters can be calculated from cross sectional OCT images (B-scans) through the optic nerve head. Optic nerve head area is the area between the boundaries of the inner limiting membrane (ILM) of the retina and Bruch’s membrane (BM) above the choroid on 2 dimensional scans, and can be interpolated across adjacent scans to calculate volume [7]. Flattening of the ocular globe can be characterized using 2 or 3-dimensional shape analysis of the Bruch’s membrane contour on either side of the optic nerve head [8].

Prior studies investigating OCT derived quantitative optic nerve head metrics of elevated optic nerves have utilized a single device, scan protocol and analysis technique. Though many systematic comparisons of OCT devices and scan protocols are available in the literature, none have yet to address the assessment of elevated optic nerves, which bring unique challenges including identification of the medial margins of Bruch’s membrane due to limited penetration of the frequency domain OCT laser through an elevated optic nerve head [3]. The present study aims to compare OCT images of swollen optic nerve heads obtained with difference devices and scan protocols and to assess reliability within raters, between raters and between devices with regards to quantitive metrics.

## Methods

Potential subjects were identified through retrospective chart review of patients seen in the neuro-ophthalmology clinic at Byers Eye Institute at Stanford where an ongoing quality improvement project includes comparison of OCT devices and scan patterns. Screening criteria were neuro-ophthalmology clinic visit for possible optic neuropathy and OCT images of the optic nerve obtained with multiple devices using both wide and narrow field scan patterns. Inclusion criteria were presence of disc swelling in at least one eye. In addition, a single subject with normal optic nerve appearance in both eyes was included. This study was approved by the Stanford University Institutional Review Board with a waiver of consent due to the retrospective nature of the study. Diagnosis was extracted from the medical record.

Scan patterns for the included subjects were performed on each of the three spectral domain (SD)-OCT devices (Cirrus HD-OCT, Carl Zeiss Meditec Inc., Dublin, CA; Avanti, Optovue Inc., Freemont, CA; OCT Spectralis, Heidelberg Engineering Inc., Heidelberg, Germany). The images collected using the Zeiss Cirrus platform were a 12-slice radial scan centered on the optic nerve head with a scan length of 6-mm and a 9-mm HD line scan (average of 100 sweeps) oriented to intersect the fovea and the center of the optic nerve. Images collected using the Heidelberg Spectralis platform were a 6-slice radial scan centered on the optic nerve head with a scan length of 30°, and 30° high resolution line scans with and without enhanced depth imaging (EDI) oriented to intersect the fovea and the center of the optic nerve. All line and radial scans taken using the Spectralis machine used high resolution settings and automated real time tracking (ART) with 100 frames. The images acquired using the Optovue Avanti platform were an 18-slice radial scan with a scan length of 6-mm, a 9-mm standard definition line scan, and a 6-mm HD line scan oriented to intersect the fovea and the center of the optic nerve. Both the enhanced and standard definition lines were taken with a scan number of 250.

**Fig. 1:**
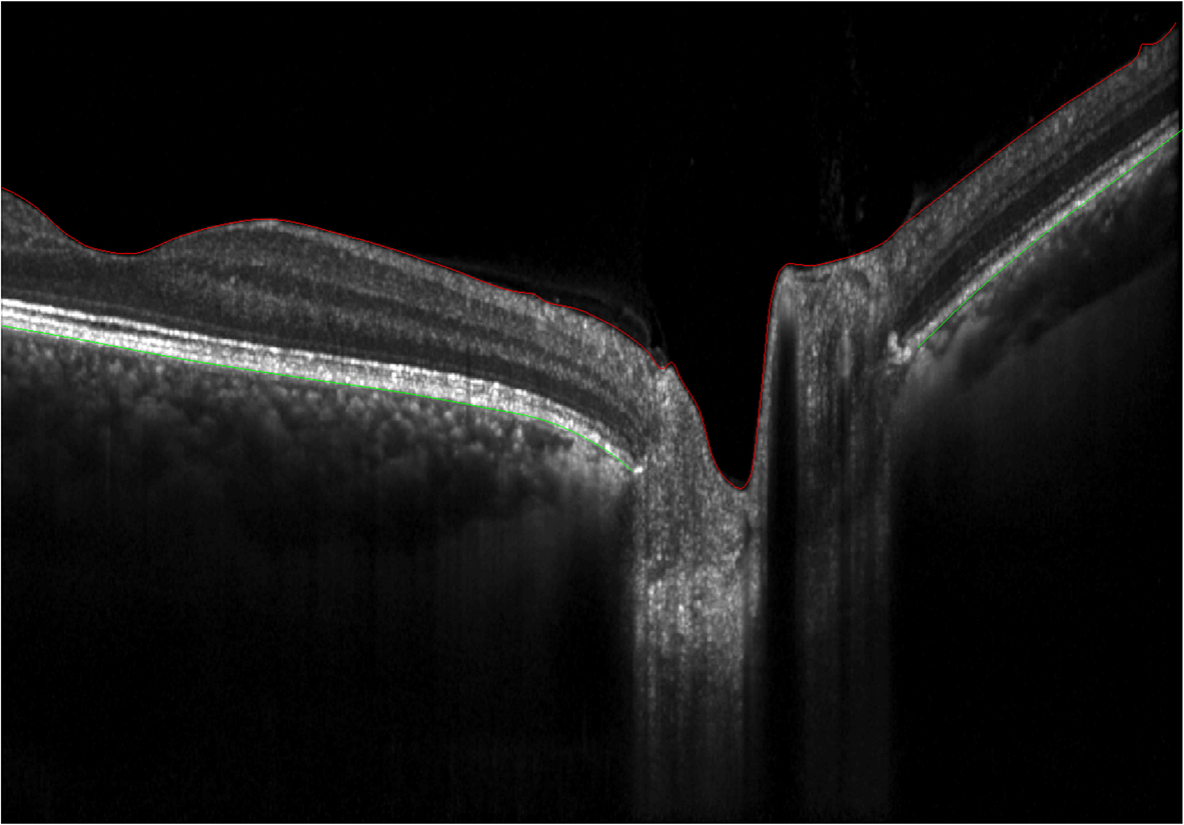
An image of a non-swollen optic nerve with semi-manual segmentations completed using custom MATLAB-based software. The ILM is shown in red and the left and right sides of BM are shown in green

Raw data from all three machines (*.img from Cirrus, *.OCT from Avanti and *.vol from Spectralis) were analyzed semi-manually using a modification of custom MATLAB-based segmentation software (A. Raza, X. Zhang, Columbia University, New York) [9]. Specifically, for each image, points defining the upper internal limiting membrane (ILM) and the temporal and nasal BM (relative to the optic nerve) were identified by a rater using a cursor. A curve fitting algorithm interpolated between rater identified points and the rater added, removed and/or redefined points to adjust the curve so that it traced the structure of interest (Fig. 1). Contrast and brightness adjustments were used at the discretion of each rater to enable identification of the boundaries of interest. Three raters independently segmented each of the scans and one rater segmented each scan twice on two separate occasions.

Qualitative feedback was collected from raters regarding ease of segmentation for each device/scan protocol. Y (axial) and X coordinates for segmentations were converted to μm using image specific scaling factors for each device. Segmentation difference between and within raters for each scan was quantified as the differences in axial location for a given segment for a given horizontal location (in pixels and microns) averaged across a single B-scan. 95% limits of agreement for axial segmentation were calculated as mean ± 1.96 * standard deviation. Outliers were those images with differences exceeding the limits of agreement. These were reviewed to identify image features prone to segmentation disagreement.

Optic nerve cross sectional area, the region between ILM and BM on nasal/temporal scans truncated to 2.85 mm on either side of the scan center, was calculated for each scan. To do this, nasal and temporal sides of BM were joined together by interpolating a straight line between the user identified margins beneath the optic nerve head to create a continuous BM boundary. Reliability of optic nerve head area between raters and within raters was assessed for each device’s radial and line scan protocols using two-way random intraclass correlation coefficients(ICC). Bland Altman plots were used to graphically assess reliability for pairwise comparisons of raters with attention to systematic variability and variability as a function of optic nerve head area. Comparison between devices was performed using similar techniques for radial scan protocols centered on the optic nerve head. Line scan comparison between devices and with radial scans were not performed due to variation in line scan positioning. Statistical analysis was performed using SPSS V.24 (IBM Inc.).

## Results

Eleven potential subjects were identified by screening. Both eyes of the five with active disc swelling and one subject with normal optic nerve appearance in both eyes were included in further analysis. Diagnoses were bilateral papilledema due to elevated intracranial pressure, bilateral optic disc drusen, unilateral anterior optic neuritis, unilateral acute non-arteritic anterior ischemic optic neuropathy and no optic nerve swelling. Thus, the images studied represented 6 swollen optic nerve heads and 4 non-swollen optic nerve heads. Cirrus signal index ranged from 4 to 10, Avanti signal index ranged from 11 to 88 and Spectralis signal to noise ratio ranged from 19 to 47 dB for radial scans. One eye of one subject had scan quality below the manufacturers’ minimum recommendations for quality for Cirrus (6) and Avanti scans (30). This eye also had the lowest Spectralis signal to noise ratio. Therefore, this eye was excluded from further analysis. All other scans exceeded minimum quality recommendations.

### Image comparison

**Fig. 2:**
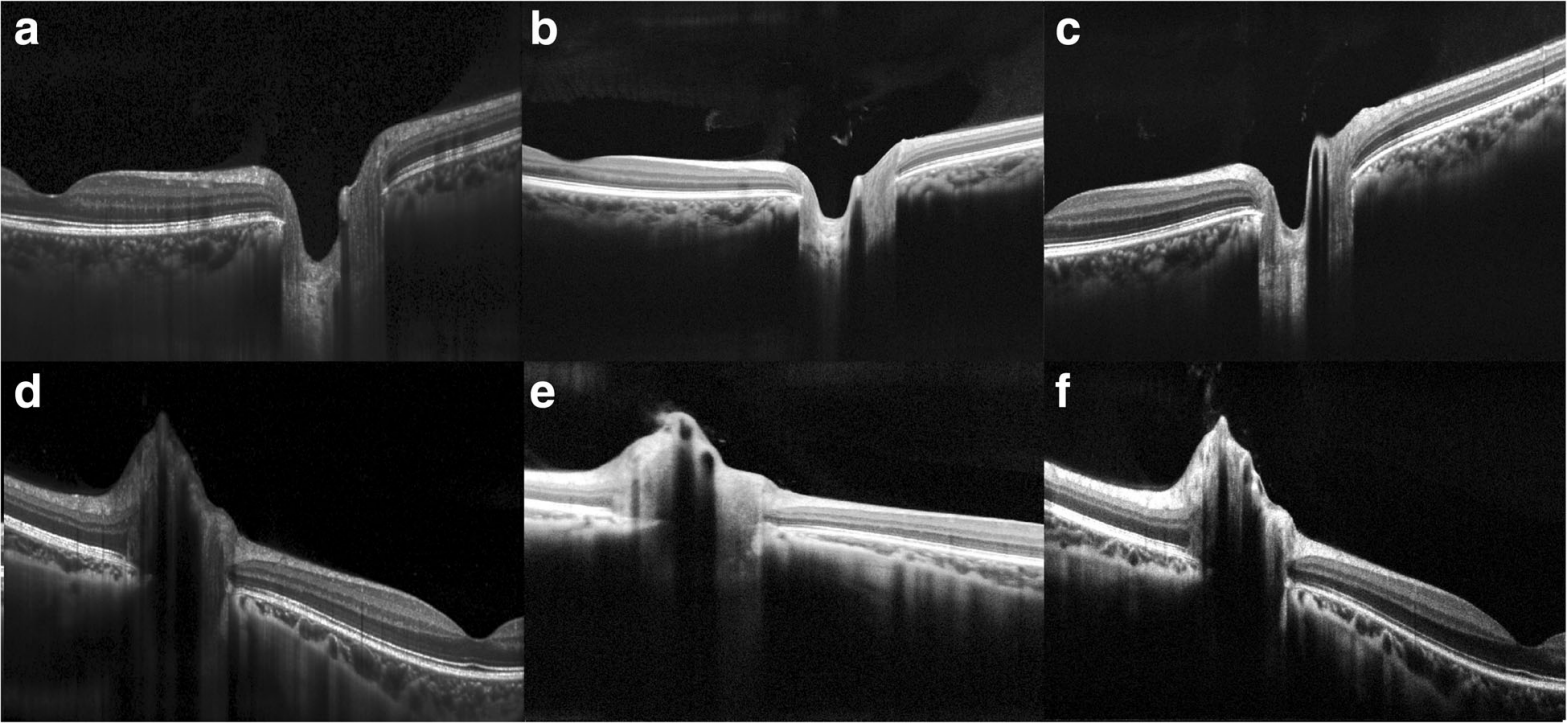
Approximately 9-mm OCT line scans oriented to intersect the fovea and the center of the optic nerve. (**a**, **d**) show high resolution line scans taken on the Heidelberg Spectralis OCT device. (**b**, **e**) show standard definition line scans acquired on the Optovue Avanti OCT device. (**c**, **f**) show high definition line scans taken on the Zeiss Cirrus OCT device. (**a**-**c**) are images of a non-swollen optic nerve while (**d**-**f**) are images of a swollen optic nerve in a subject with papilledema due to elevated intracranial pressure

Line scans from the three OCT acquisition devices were qualitatively similar in terms of ILM and BM visibility for both swollen and non-swollen optic nerves (Fig. 2). Raters reported similar ease of segmentation for both the ILM and BM on line scans from all devices with little subjective difficulty in determining the BM medial margins in non-swollen eyes. In swollen eyes, raters reported similar difficulty in identifying BM medial margins across line scans from all 3 OCT devices. Raters noted that variations in appearance of the ILM-vitreous interface and the outer retina-BM interface were slightly different between devices leading to some uncertainty regarding the ILM location.

On the Spectralis platform line scans performed with EDI had no significant qualitative effect on the rater reported distinction of BM margins beneath the optic nerve head. However, scans with EDI had noticeably decreased resolution of the optic nerve head surface in swollen nerves, impacting segmentation of the ILM. On the Avanti platform the enhanced high definition line scan did not differ subjectively from the standard definition scan with regards to ease of segmentation of the ILM and BM boundaries.

**Fig. 3:**
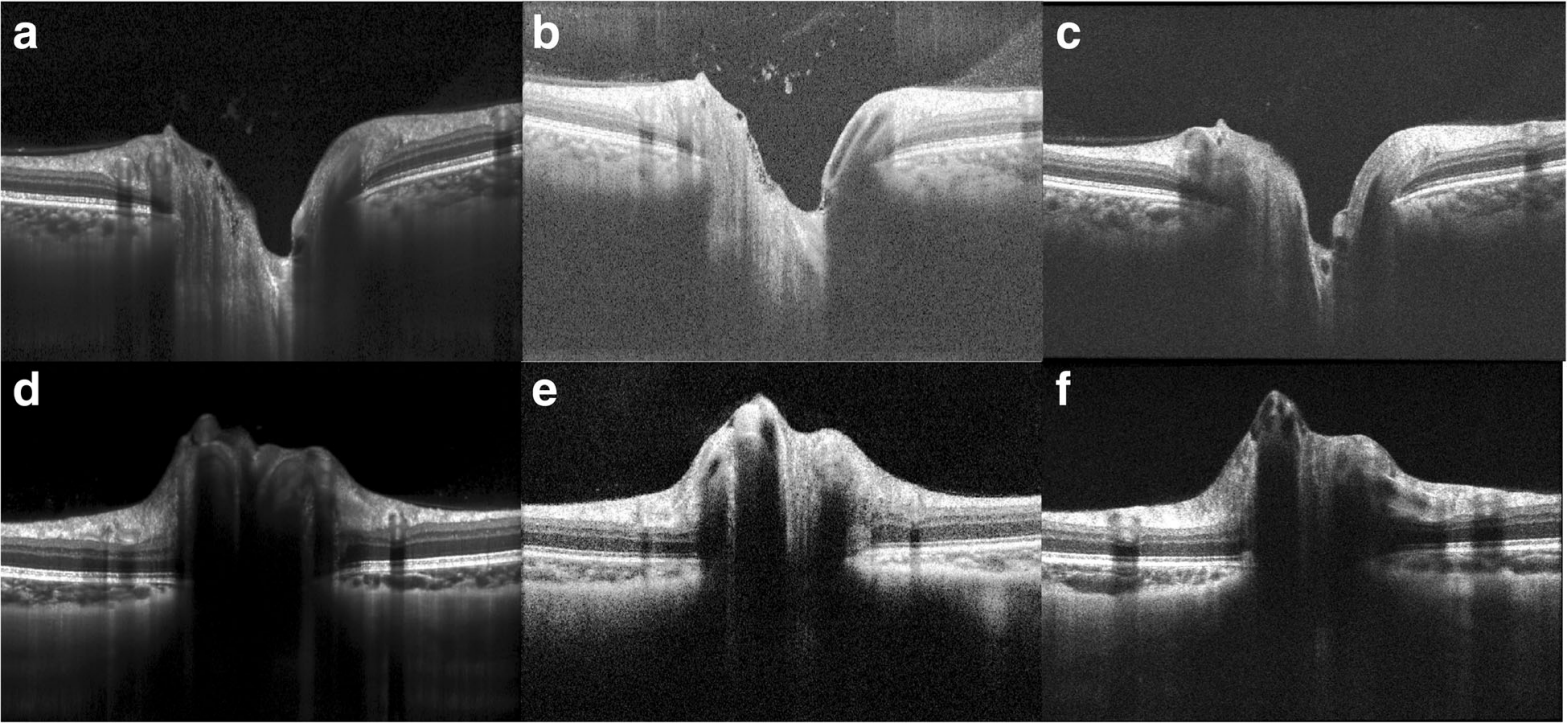
Approximately 6-mm OCT scans centered over the optic nerve (taken from radial scan patterns). (**a**, **d**) show radial scans taken on the Heidelberg Spectralis OCT device. (**b**, **e**) show radial scans acquired on the Optovue Avanti OCT device. (**c**, **f**) show radial scans taken on the Zeiss Cirrus OCT device. (**a**-**c**) are images of a non-swollen optic nerve while (**d**-**f**) are images of a swollen optic nerve in a subject with papilledema due to elevated intracranial pressure

Radial scans differed from the line scans with regards to the ease of identifying and segmenting ILM and BM (Fig. 3). Overall, raters found the radial scans were more difficult to segment than the high definition line scans with the radial scan most closely matching the clarity of the line scan for the Spectralis device.

### Segmentation comparison

**Fig. 4:**
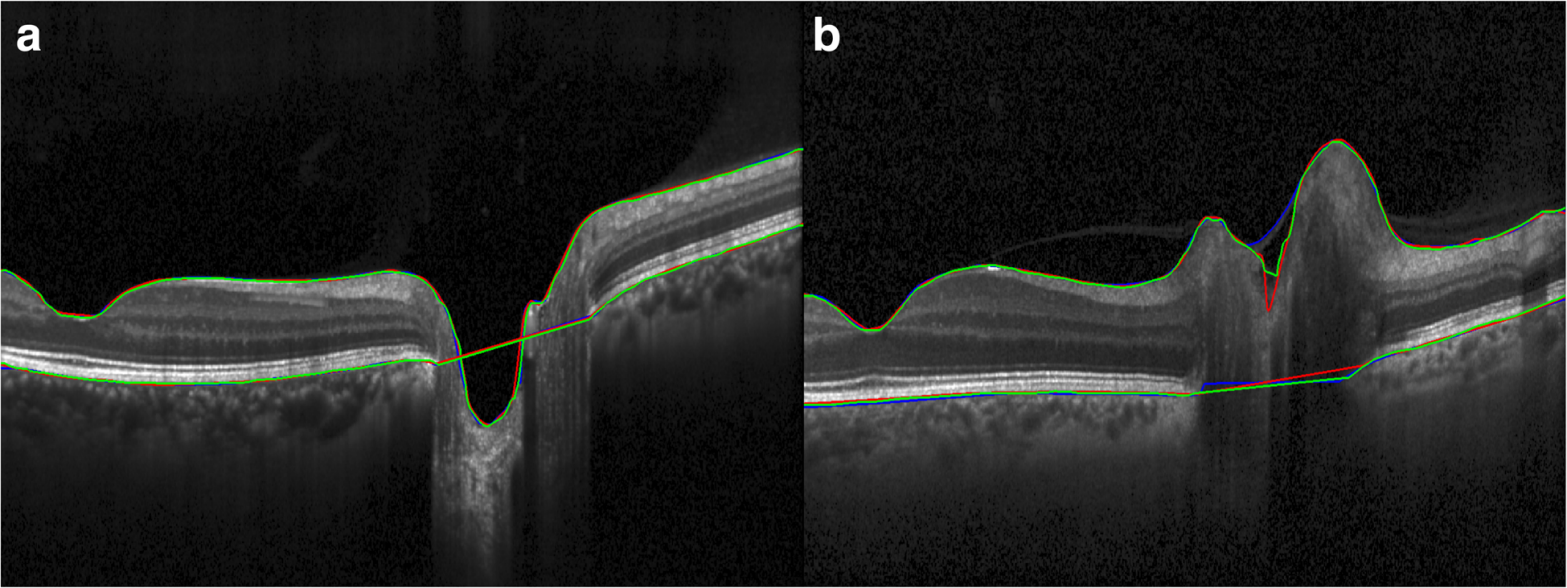
Inter-rater segmentation variability (**a**) shows a high-resolution 30° line scan of a non-swollen optic nerve taken on the Heidelberg Spectralis OCT device. **b** shows a high-resolution 30° line scan of a swollen right eye optic nerve (papilledema) taken on the Heidelberg Spectralis OCT device. Both images also show independent segmentations of the ILM and BM done by 3 different raters (red, blue, green). The raters are generally in better agreement in segmenting the non-swollen optic nerve (**a**) when compared to the swollen optic nerve (**b**). Panel b shows the disagreement in ILM segmentation within the cup of the optic nerve due to a possible artifact. Panel b also shows that inconsistent identification of the medial margins of BM results in differences in the interpolated line connecting the left and right segments of BM

Inter-rater differences across scan types and devices (averaged for each scan) were 7.8 ± 3.6 μm in the axial dimension for ILM segmentation and 10.7 ± 4.1 μm in the axial dimension for BM segmentation. Three outliers above the upper boundary of the 95% limit of agreement of 14.9 μm for ILM and one outlier with above the upper boundary of the 95% limit of agreement of 18.6 μm for BM occurred in different eyes of different subjects. These were attributable to differences in rater selection of the segmentation boundary (e.g. segmentation of vitreous interface instead of ILM). ILM in the region of the cup and BM beneath the optic nerve were regions most subject to disagreement between raters (Fig. 4).

Intra-rater differences across scan types and devices (averaged for each scan) were 3.6 ± 0.96 μm in the axial dimension for ILM segmentation and 4.1 ± 2.7 μm in the axial dimension for BM segmentation. There were a single ILM outlier with average difference above the upper boundary of the 95% limit of agreement of 5.4 μm and a single BM outlier above the upper boundary of the 95% limit of agreement of 9.7 μm. The latter occurred in the same eye that was a BM outlier for inter-rater differences and was attributable to a different user choice in identification of the BM segment.

### Optic nerve cross sectional area comparison

**Fig. 5:**
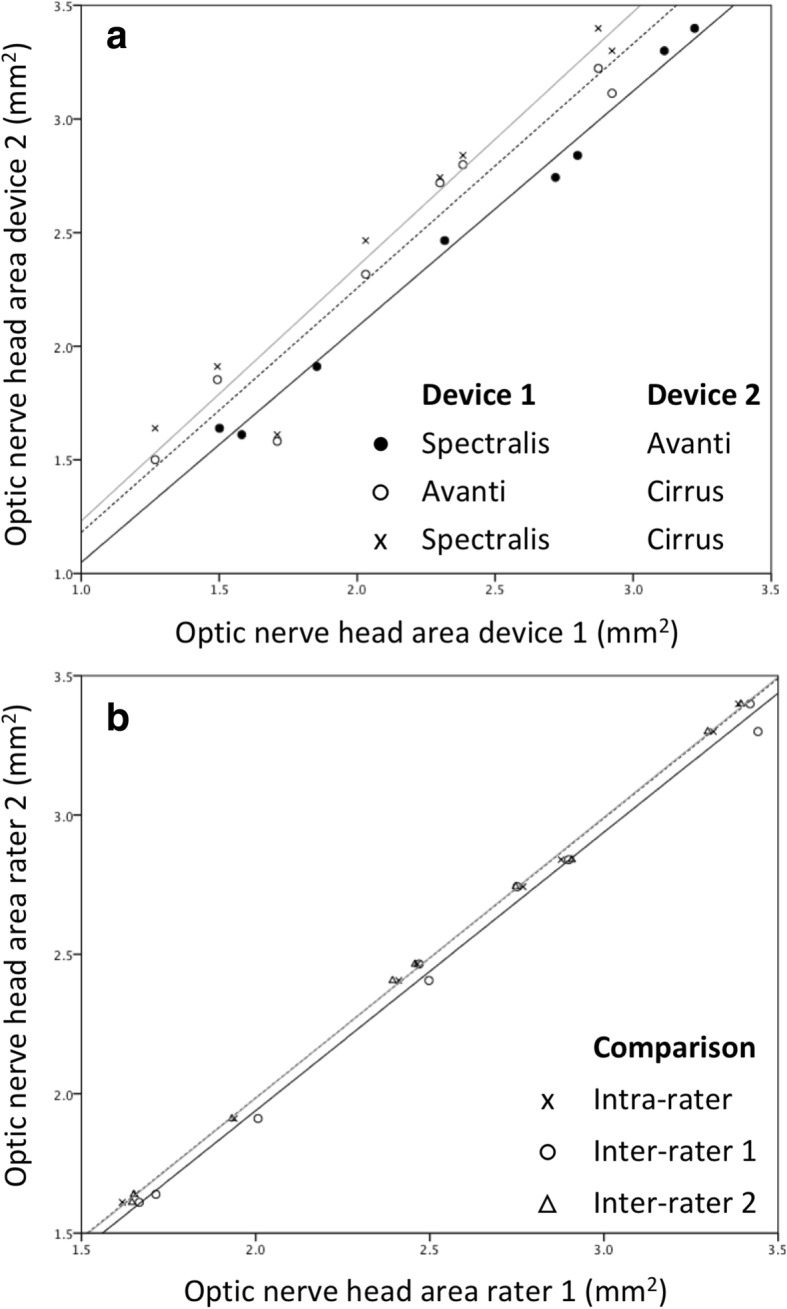
Comparison of optic nerve head cross sectional area calculated using images from different devices (**a**) and using segmentations by different raters (**b**). Distinct comparisons are indicated by marker type. Lines are best fit linear regression

Optic nerve cross sectional area in a 5.7 mm diameter nasal-temporal scan centered on the optic nerve head (radial scan protocol) ranged from 1.6 to 3.4 mm^2^. Measurements based on different rater segmentations and from images taken with different devices were similar with larger differences between devices than between rater (Fig. 5). The maximum intra-rater difference (0.08 mm^2^) was less than the maximum inter-rater difference (0.14 mm^2^), which was less than the maximum inter-device difference (0.58 mm^2^). Intraclass correlation coefficients (ICC) for optic nerve area derived from radial scan protocols were excellent for intra-rater, inter-rater and inter-device comparisons with the latter being slightly lower (Table 1). Inter-rater and intra-rater ICC for optic nerve cross sectional areas from line scans were similar to those for radial scans, ranging from 0.999 to 1.00.

**Table 1:** Intra-class correlation coefficients for absolute agreement of optic nerve cross sectional area calculated OCT B-scans centered on the optic nerve head

	Absolute agreement
Intra-rater (*n* = 2)	0.999–1.00 (3 devices)
Inter-rater (*n* = 3)	0.999–1.00 (3 devices)
Inter-device (*n* = 3)	0.958–0.965 (3 raters)

**Fig. 6:**
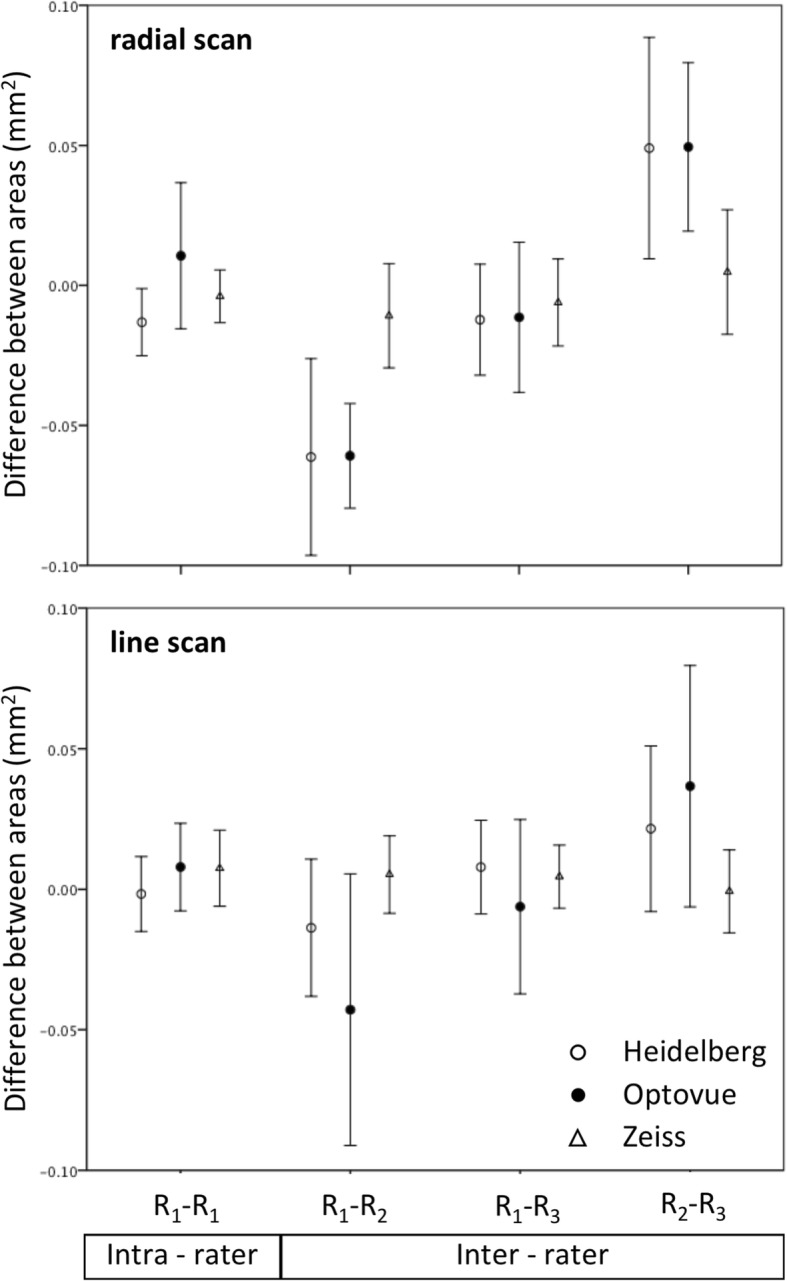
Ninety five percent limits of agreement for optic nerve head cross sectional area derived from radial (upper) and line (lower) scan protocols analyzed by different raters stratified by acquisition device. Each marker represents the mean difference between scans rated twice by the same rater (intra-rater) and by three different raters (inter-rater). Error bars represent 95% confidence interval for the comparison

**Fig. 7:**
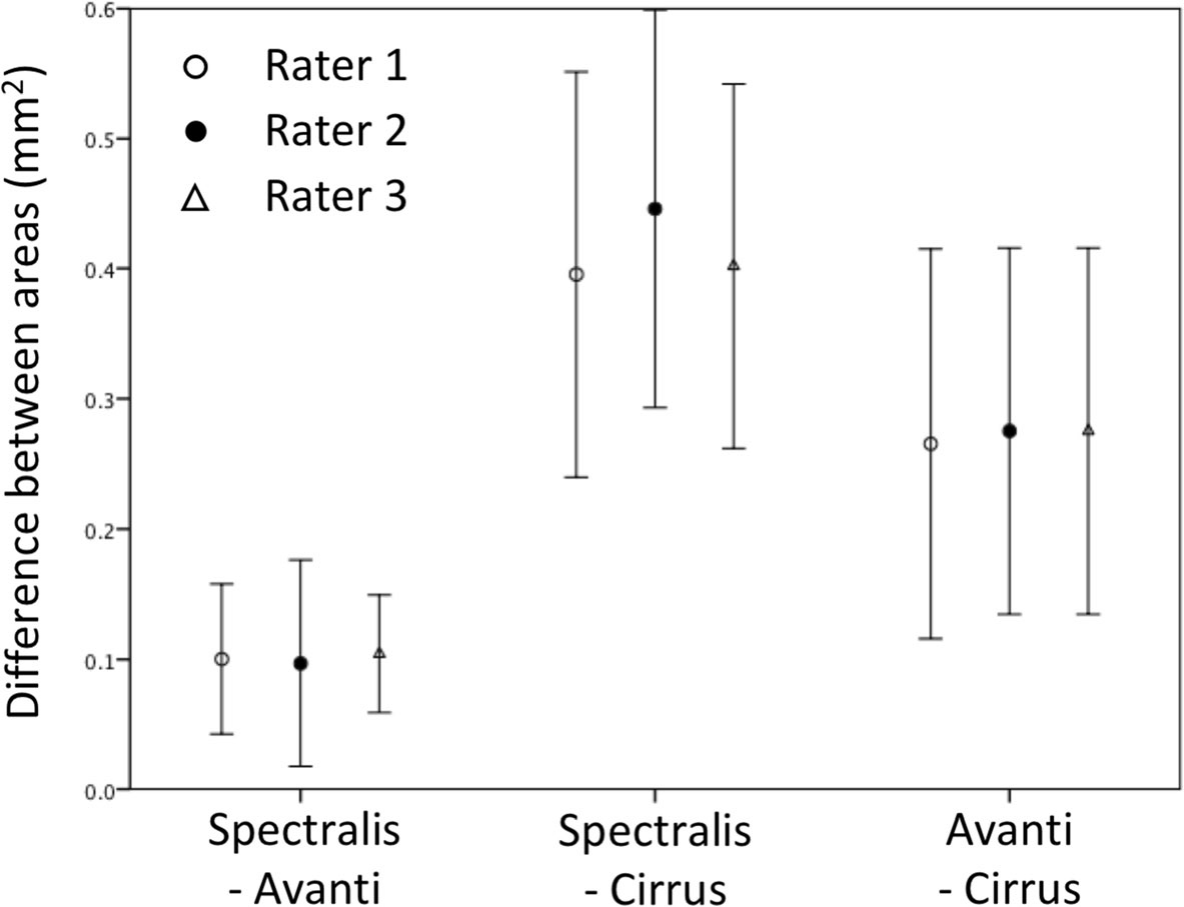
Ninety five percent limits of agreement for optic nerve head cross sectional area derived from radial scan protocols for different acquisition device stratified by rater. Each marker represents the mean difference between scans from two different devices. Error bars represent 95% confidence interval for the comparison

Intra-rater 95% limits of agreement for optic nerve head area derived from radial scans had less systematic error (i.e. distance of mean from 0) and were narrower than for inter-rater agreement (Fig. 6a). The systematic error for inter-rater agreement was less for the line scan derived area than for radial scan derived area (Fig. 6b). Inter-device 95% limits of agreement had larger systematic error and were wider than both intra and inter-rater agreement (Fig. 7). Bland Altman plots for each two-way comparison did not show differences or outliers to be related to measurement level.

## Discussion

Though there is extensive literature regarding development of OCT extracted biomarkers of ONHV and globe flattening for diagnosis and monitoring of anterior optic nerve disorders and ICP, and extensive literature comparing OCT devices and scan protocols, to our knowledge, there has not previously been a direct comparison of scan patterns and acquisition devices for imaging elevated optic nerves. In this paper, we contribute a comparison of wide angle line scans through the fovea and optic nerve with narrower angle scans through the optic nerve head obtained using SD-OCT devices from three different manufacturers analyzed by three different raters. The results have relevance for selecting scan protocols from which to derive OCT based measures of swollen optic nerve heads.

On a qualitative basis, the wide angle line scans were similar across devices with good definition of ILM and peripheral BM and similar shadowing beneath the swollen optic nerve heads. Scans obtained using radial protocols were of lower quality which made segmentation subjectively more challenging. The Heidelberg Spectralis radial scans most closely matched the quality of the wide angle line scans. This may be attributed to the fact that the Spectralis native software allows for users to adjust the ART frames on the radial scan protocol, whereas the other two platforms have fewer options for user customization of the radial scan protocol.

None of the SD-OCT devices or scan patterns eliminated the challenge that uniquely affects evaluation of swollen optic nerves, namely identification of BM margins below a swollen optic nerve. The Spectralis EDI option did not improve identification of BM margins, but reduced rater confidence in segmenting ILM. Swept source (SS) OCT may allow for better visualization of the medial margins of BM due to increased penetration through the swollen optic nerve tissue that occurs due to using a light source with a longer central wavelength (λ) than the that of the SD-OCT devices used in this study. However, because axial resolution is proportional to λ^2^/Δλ, where Δλ is the bandwidth, the longer central wavelength may be associated with worse axial resolution if bandwidth is not proportionally larger. Published estimates of commercially available SS and SD OCT suggest that axial resolution is slightly better for SD-OCT (7 μm for Spectralis OCT2 used in this study vs. 8.1 μm) [10, 11]. This may reduce precision of imaging-based estimates. Another approach might be to address the problem of BM shadowing analytically, for example by excluding regions of the image prone to this artifact from analysis.

As expected, differences in segmentation were less for intra than inter-rater comparisons. Inter-rater disagreement for segmentation was greater for BM than ILM across acquisition devices. This is likely because image quality deteriorates with depth in OCT and adjacent hyperdense structures in the choroid can impede interpretation of the BM contour.

For optic nerve area measurement, intra-rater differences were smaller and with less systemic error when compared to the inter-rater differences. This can likely be attributed to consistent judgement regarding segments by an individual and stems from the smaller segmentation differences. It suggests that inter-rater agreement might be improved upon by training sessions and consensus review of segmentations. For example, a training set of images of both normal and swollen eyes due to a variety of optic neuropathies could be used to calibrate raters regarding identifying the different structures of the optic nerve, discerning retinal tissue from artifacts, and more confidently and consistently identifying BM margins. Inter-rater agreement showed less systematic error for wide angle line scans than for radial scans which might be due to better scan quality enabling easier and more consistent judgements regarding location of boundaries during segmentation.

Inter-device differences were greater and with more systemic error than inter-rater differences. The random error may be due to differences in scan positioning and the systematic error due to calibration of each device. As with other quantitative OCT measures, this suggests that comparison between metrics obtained with different devices is not advisable without accounting for systematic error.

Though this study used a semi-manual segmentation protocol, the issues identified are not unique to this methodology. Challenges of artifacts and shadowed BM are also a challenge for automatic segmentation algorithms to the extent that many use a semi-manual identification of the BM margins to seed the automatic algorithm [3]. It is imperative that any automatic algorithm be tested on a set of images that captures typical artifacts.

## Conclusions

The present study characterizes the effects of segmentation raters, OCT acquisition device and scan pattern on images, segmentation, and segmentation derived values of optic nerve heads with varying degrees of elevation. It highlights important considerations when selecting a scan protocol and segmentation strategy for calculation of optic nerve head structural parameters from OCT images. Further study is needed to characterize the differences due to repeat imaging (e.g. test, retest) and to determine the clinical threshold for error.

## Abbreviations

ART: Automated real-time tracking
BM: Bruch’s membrane
EDI: Enhanced depth imaging
ICP: Intracranial pressure
ILM: Inner limiting membrane
OCT: Optical coherence tomography
ONHV: Optic nerve head volume
SD: Spectral domain
SS: Swept source
